# Asymmetric Near-Complete Ossification of Reichert’s Cartilage

**DOI:** 10.7759/cureus.108161

**Published:** 2026-05-03

**Authors:** Maria Piagkou, Panayiotis Papadopoulos, Nikolaos-Achilleas Arkoudis, Nikolaos Lazaridis, George Triantafyllou

**Affiliations:** 1 Department of Anatomy, School of Medicine, Faculty of Health Sciences, National and Kapodistrian University of Athens, Athens, GRC; 2 Second Department of Radiology, School of Medicine, University General Hospital of Athens "Attikon" National and Kapodistrian University of Athens, Athens, GRC; 3 Department of Anatomy and Surgical Anatomy, School of Medicine, Faculty of Health Sciences, Aristotle University of Thessaloniki, Thessaloniki, GRC

**Keywords:** anatomical variation, carotid artery compression, computed tomography angiography, hyoid bone, ossification, reichert’s cartilage, stylohyoid chain, styloid process

## Abstract

This report describes a rare case of asymmetric bilateral ossification of Reichert’s cartilage (RC), identified incidentally in a male patient. During a retrospective imaging study of a Greek population, the computed tomography angiography (CTA) of a 43-year-old man was selected due to its unusual morphology. Near-complete asymmetric ossification of the RC was observed, coexisting with an anisomorphic and asymmetric hyoid bone (HB). The right and left greater horns (GHs) measured 28.21 mm and 27.07 mm, respectively. Only the right lesser horn (LH) was identified, which demonstrated ossification and measured 12.17 mm. The left styloid process (SP) measured 78.6 mm in length and 13.81 mm in maximum width, with an angulation of 69.91°, demonstrating near-complete ossification extending to the upper border of C3. It was closely related to the internal carotid artery (ICA) and external carotid artery, with focal compression of the ICA at the superior margin of C2. The right SP measured 53.7 mm in length and 6.54 mm in width, with an angulation of 56.26°. Distal ossification of the RC was also present, arising 14.1 mm from the HB. Additionally, the linguofacial trunk was identified. Recognition of such morphological variants of the RC and HB may improve clinical assessment and surgical planning in the cervical region. Three-dimensional volume-rendered images and multiplanar reconstructions are strongly recommended for accurate anatomical evaluation.

## Introduction

Reichert’s cartilage (RC) contributes to several skeletal and connective tissue structures of the head and neck. Traditionally, it is divided into four segments: (1) the tympanohyal, which contributes to the proximal styloid region and attachment to the stapes; (2) the stylohyal, which forms the styloid process (SP); (3) the ceratohyal, which develops into the stylohyoid ligament (SHL), extending from the tip of the SP to the lesser horn (LH) of the hyoid bone (HB); and (4) the hypohyal, which contributes to the LH and body of the HB. However, some embryological studies suggest that these divisions may be less discrete than previously described, supporting a more continuous developmental pattern. This concept may be relevant to understanding craniofacial variants and abnormalities of HB morphology [1,2].

The SP, SHL, and LH together form the stylohyoid chain, also referred to as the stylohyoid complex (SHC). The term SHC syndrome has been proposed to describe painful lateral cervical or craniofacial conditions associated with an elongated SP (ESP), partial or complete ossification of the SHL, and/or elongation of the hyoid apparatus [1,2].

The RC segment between the SP and the LH normally gives rise to the SHL and corresponds to the ceratohyal portion [3]. In human embryological studies, Rodríguez-Vázquez et al. did not observe the classic four-part segmentation of the SHC; instead, they identified a stylohyal component in continuity with the hypohyal segment [1].

An SP is generally considered elongated when it exceeds 30 mm in length. In our recent meta-analysis, the pooled prevalence of unilateral ESP was 25.03%, whereas bilateral ESP was observed in 16.04% of individuals [4]. Despite this relatively common anatomical finding, only a small proportion of affected individuals (approximately 4%) become symptomatic [4,5]. In bilateral cases, symptoms are often unilateral [5]. The SHC is located within a “high-traffic” anatomical corridor of the neck, acting as a critical crossroads for vital neurovascular structures [1-5]. Due to the density of essential structures in this confined space, even minor bony deviations or subtle ossifications within the SHC can exert significant mechanical effects on the internal and external carotid arteries [1-5]. Factors associated with Eagle syndrome include elongation or increased anterior angulation of the SP, as well as ossification of the SHL or stylomandibular ligament [6,7]. Clinically, Eagle syndrome most commonly presents with dysphagia, foreign body sensation in the throat, and pain involving the oropharyngeal, cervical, or craniofacial regions, often exacerbated by swallowing, tongue movement, yawning, or head rotation [5].

This report describes incidentally detected asymmetric bilateral ossification of the RC identified on computed tomography angiography (CTA) in a male patient. In addition, asymmetry of the HB morphology was noted. This case highlights the possible contribution of HB morphological variation to bilateral RC ossification.

## Case presentation

During a retrospective imaging study of a Greek population, the CTA of a 43-year-old male patient was selected for further evaluation due to its unusual morphology. The patient had been admitted to the hospital for severe COVID-19 infection, and his medical history was not related to the anatomical findings described. The images were acquired using a 128-slice multidetector computed tomography scanner (SOMATOM go.Top, Siemens, Malvern, PA, USA) at the General Hospital of Nikaia-Piraeus, with a slice thickness of 0.8 mm. The patient was positioned supine with the head in a neutral position. Imaging was performed following injection of 60 mL of a 30% iodine contrast solution at a flow rate of 4-4.5 mL/s. The data were analyzed and documented using Horos software (version 3.3.6; Horos Project, New York, NY, USA). Measurements of the SP, including the length, width, and angulation, were obtained using pre-established protocols [6,7]. Near-complete asymmetric ossification of RC was identified, associated with an anisomorphic and asymmetric HB.

**Figure 1:**
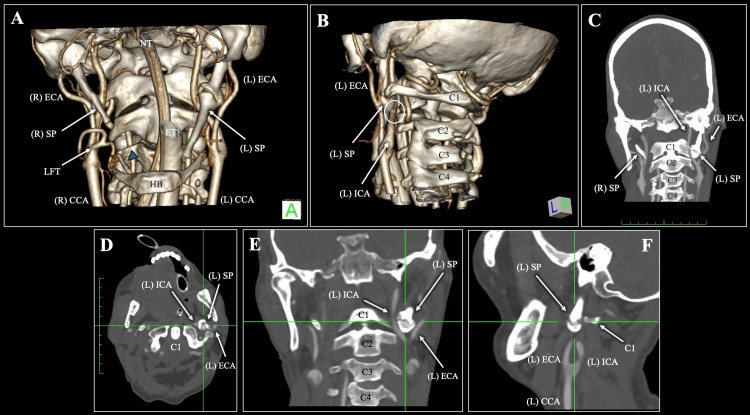
Three-dimensional reconstructions (A, B), maximum-intensity projection (C), and multiplanar reconstructions (D-F) of the patient’s multidetector computed tomography angiogram (CTA), demonstrating bilateral asymmetric ossification of Reichert’s cartilage. The blue arrowhead in (A) indicates the distal ossified segment of the right styloid process. (A) Anterior view. (B) Left lateral/posterior oblique view. (C) Coronal maximum intensity projection. (D) Axial reconstruction. (E) Coronal reconstruction. (F) Sagittal reconstruction. CCA: common carotid artery, ECA: external carotid artery, ICA: internal carotid artery, SP: styloid process, HB: hyoid bone, LFT: linguofacial trunk, ET: endotracheal tube, NT: nasogastric tube, L: left, R: right, C1: atlas, C2: axis.

The left SP was markedly elongated and enlarged, measuring 78.6 mm in length, with a maximum width of 13.81 mm and an angulation of 69.91°. It extended from the temporal bone toward the HB, forming near-complete ossification of the RC up to the upper border of the third cervical vertebra (C3) (Figure 1). At its point of maximal width, the ossified structure was closely related to the internal carotid artery (ICA), external carotid artery (ECA), and trachea. Notably, it caused focal compression of the ICA at the superior margin of the second cervical vertebra (C2) (Figure 1). The ICA diameter measured 4.3 mm proximal to this point and decreased to 2.7 mm at the level of compression, representing a 43.8% reduction.

The right SP was elongated to the level of the upper margin of C2, measuring 53.7 mm in length, with a maximum width of 6.54 mm and an angulation of 56.26°. Distal ossification of the RC arising from the HB measured 14.1 mm in length (Figure 1). A close anatomical relationship was observed between the ossified RC variant and both the ICA and ECA (Figure 1). A linguofacial trunk (LFT) was also identified (Figure 1).

The LH of the HB was not visualized on the left side. The elongated greater horns (GHs) measured 27.07 mm on the left and 28.21 mm on the right. Notably, ossification was observed only in the right LH, which measured 12.17 mm in length. The height of the HB body measured 19.58 mm.

This report is limited by the inability to correlate the near-complete asymmetric ossification of RC with the anisomorphic HB morphology and its potential clinical manifestations.

## Discussion

According to Rodríguez-Vázquez et al. [1], variability in the form and length of the cranial segment of RC may give rise to a SP of variable length and inclination. Lengele and Dhem [8] demonstrated that both long and short SPs may share characteristics of calcified cartilage. The anatomical relationships of the angulated inferior end of the cranial (styloid) segment of RC may help explain the common symptoms associated with Eagle syndrome [5]. Rodríguez-Vázquez et al. [1] suggested that dysphagia and a foreign body sensation in the throat may result from the tip of an angulated SP lying close to the pharyngeal wall. Accordingly, Frommer [9] proposed that SP direction and curvature may be more clinically relevant than length alone. Sore throat, pain radiating along the distribution of the glossopharyngeal nerve [9], and even taste disturbances [3] may be explained by the close anatomical relationship between this nerve and RC. According to Graf [10], during swallowing, the glossopharyngeal nerve may be compressed against an osseous spicule, producing paroxysmal pain.

The pathogenesis of elongation and mineralization within the SHC remains incompletely understood. Some theories suggest that congenital elongation of the SP results from persistence of cartilaginous tissue within the stylohyal segment, combined with calcification of the SHL. In adults, the SHL, typically composed of dense fibrous connective tissue, may retain remnants of embryonic cartilage that later undergo partial or complete ossification [11,12].

Several hypotheses have been proposed to explain abnormal ossification in patients with elongated SPs, including reactive hyperplasia, reactive metaplasia, anatomical variation, age-related developmental anomalies, genetic predisposition, and endocrine dysfunction in postmenopausal women [5]. Another theory attributes SP elongation to persistence of embryonic tissue derived from RC [1]. Variations of the hyoid apparatus may therefore represent developmental variants of RC. If these changes persist into adulthood, they may help explain the symptoms associated with Eagle syndrome [1].

Symptoms may arise when ossified structures irritate adjacent tissues, including the carotid arteries and cranial nerves VII, IX, and X. Over time, gradual loss of elasticity in surrounding soft tissues may further contribute to symptom development. Reduced elasticity may also allow posterior displacement of the GH of the HB, bringing it closer to adjacent neurovascular structures [5]. Near-complete asymmetric ossification of RC may present significant intraoperative challenges. From a surgical perspective, marked elongation and increased width of the SP may distort standard anatomical planes, increasing the risk of glossopharyngeal nerve injury during tonsillectomy and vascular injury to the linguofacial trunk during submandibular gland excision. From an anesthetic perspective, rigidity of the SHC may restrict cervical extension, complicating direct laryngoscopy and increasing the risk of SP fracture or ICA compression during patient positioning [2,5].

Andrei et al. investigated SP parameters that may influence the occurrence of styloid syndromes. SP length, shape, and curvature may all be clinically relevant. CT is well suited for evaluating these features, particularly when MPRs are used. The transverse angulation of the SHC has been reported to range from 52.1° to 85.6° [13]. It has been proposed that an SP with a narrow angle (<65°) may cause symptoms by compressing adjacent structures [13]. Accordingly, in the present case, the left side demonstrated a wider angle, whereas the right side showed a narrower angle based on this threshold. In a later study, Andrei et al. [14] identified 31 SPs with narrow angulations, of which 21 were of normal length. In Eagle syndrome, medial deviation of the SP may become symptomatic by irritating the lateral pharyngeal wall. Similarly, an elongated SP associated with a narrow anterior sagittal angle may irritate the glossopharyngeal nerve within the parapharyngeal space. However, Andrei et al. [14] found only a weak positive correlation between SP length and sagittal angle, suggesting that sagittal incurvation may be of limited clinical importance. No correlation was observed between transverse angulation and SP length.

This report has several limitations. First, it describes a single incidental case; therefore, the findings cannot be generalized to the broader population. Second, the patient’s clinical history was limited, and no symptoms specifically attributable to the SHC were documented, preventing clinicoradiological correlation. Third, functional assessment of swallowing, phonation, or cranial nerve involvement was not available. Future studies should therefore include a systematic assessment of clinical symptoms using validated questionnaires. Finally, although CTA provided excellent anatomical detail, histological confirmation of the ossified structures was not possible.

## Conclusions

This case demonstrates an unusual pattern of asymmetric bilateral ossification of RC associated with anisomorphic HB morphology. The findings support the concept that developmental variations of the SHC and hyoid apparatus may persist into adulthood and contribute to rare anatomical presentations. Recognition of such variants is clinically important for radiologists, head and neck surgeons, and clinicians evaluating cervicofacial pain, dysphagia, or Eagle syndrome. High-resolution cross-sectional imaging plays a key role in identifying these abnormalities and in clarifying their relationship with adjacent neurovascular structures.
